# Moisture Source and Diet affect Development and Reproduction of *Orius thripoborus* and *Orius naivashae*, two Predatory Anthocorids from Southern Africa

**DOI:** 10.1673/031.012.0101

**Published:** 2012-01-09

**Authors:** Jochem Bonte, Dominiek Vangansbeke, Sara Maes, Maarten Bonte, Des Conlong, Patrick De Clercq

**Affiliations:** ^1^ Laboratory of Agrozoology, Department of Crop Protection, Ghent University, Coupure Links 653, B-9000 Ghent, Belgium; ^2^ South African Sugarcane Research Institute, Private Bag X02, Mount Edgecombe, 4300, South Africa; ^3^ School of Biological and Conservation Sciences, University of KwaZulu-Natal, Private Bag X01, Scottsville, Pietermaritzburg, 3209. KwaZulu-Natal, South Africa

**Keywords:** Anthocoridae, biological control, factitious prey, Hemiptera, pollinivory, South Africa, zoophytophagy

## Abstract

The effect of moisture source and diet on the development and reproduction of the pirate bugs, *Orius thripoborus* (Hesse) and *Orius naivashae* (Poppius) (Hemiptera: Anthocoridae) was examined in the laboratory. Both species had been collected in and around sugarcane fields in South Africa. Supplementing eggs of the flour moth *Ephestia kuehniella* (Zeller) (Lepidoptera: Pyralidae) with a green bean pod as a moisture source yielded better nymphal survival and faster development, as compared with free water encapsulated in Parafilm, suggesting that the predators may extract extra nutrients from the bean pod. The impact of two factitious foods and moist honey bee pollen on developmental and reproductive parameters of both predators was also investigated. The overall performance of both *Orius* species on *E. kuehniella* eggs and cysts of brine shrimp, *Artemia franciscana* Kellogg (Crustacea: Artemiidae) was better than on pollen. Nonetheless, a pollen diet alone allowed 66 and 78% of the nymphs of *O. thripoborus* and *O. naivashae*, respectively, to reach adulthood. Overall, developmental and reproductive performance of *O. thripoborus* on the tested diets was superior to that of *O. naivashae.* The implications of these findings for the mass production of these predators and their potential role in biological control programs in southern Africa are discussed.

## Introduction

In southern Africa, thrips are key pests in major crops like sugarcane, citrus and avocado. Economically important thrips pests include the sugarcane thrips *Fulmekiola serrata,* the citrus thrips *Scirtothrips aurantii* and two avocado related thrips species, *Heliothrips haemorrhoidalis* and *Selenothrips rubrocinctus* (Hesse 1940; Dennil 1992; Way et al. 2006). Moreover, EPPO (2006) reported the presence in South Africa of *Frankliniella occidentalis,* a worldwide pest on a wide range of vegetable and ornamental crops. Because of their cryptic lifestyle and fast development of resistance to pesticides, thrips are difficult to control. Therefore, the availability of an effective indigenous biological control agent could provide local growers with an alternative pest management strategy.

Species of the genus *Orius* are important natural enemies of thrips and other pest species in a variety of agricultural and horticultural crops (Hernández and Stonedahl 1999) and have been widely used in biological control programs in Europe, the Americas and Asia (van den Meiracker and Ramakers 1991; Cranshaw et al. 1996; van Lenteren et al. 1997; Ohta 2001). Little is known, however, on the ecology and biocontrol potential of the *Orius* spp. native to the African continent. The pirate bugs, *Orius thripoborus* (Hesse) and *Orius naivashae* (Poppius) (Hemiptera: Anthocoridae) are commonly found in East African cropping systems (Hernández and Stonedahl 1999). Whereas *O. naivashae* was only known from Kenya (Hernández and Stonedahl 1999), *O. thripoborus* has been found in St. Helena (Carayon 1976), South Africa (Hesse 1940), and Kenya (van den Berg and Cock 1995), but is probably more widely distributed in intervening parts of southern Africa (Hernández and Stonedahl 1999). Dennil (1992) suggested that *O. thripoborus* might be a useful biological control agent against *H. haemorrhoidalis* and *S. rubrocinctus* in the eastern Transvaal. In order to optimize and rationalize the mass production and release of these predators, it is crucial to understand their nutritional ecology. Important aspects of a successful mass rearing system for these *Orius* spp. include the availability of alternative food sources, adequate water sources, and suitable oviposition substrates.

Plant feeding has been shown to provide moisture and nutrients to numerous predatory heteropterans (Kiman and Yeargan 1985; Ruberson et al. 1986; Legaspi and O'Neil 1993; Coll 1996, 1998; Armer et al. 1998; Lundgren 2009). Additionally, *Orius* spp. are zoophytophagous, allowing them to benefit from plant materials and animal prey (Coll 1996; Lattin 1999). Several *Orius* spp. are able to develop on certain pollens as a sole food source (Kiman and Yeargan 1985; Vacante et al. 1997; Venkatesan et al. 2008).

In commercial insectaries, *Orius* bugs are mainly reared on eggs of the Mediterranean flour moth *Ephestia kuehniella* (Zeller) (Lepidoptera: Pyralidae), which constitutes an effective but expensive factitious (i.e. unnatural) food. This has resulted in a search for cheaper alternative foods, such as brine shrimp (*Artemia* sp.) cysts (Arijs and De Clercq 2001; De Clercq et al. 2005) and various artificial diets (Zhou and Wang 1989; Cohen 1999; White et al. 2000; Arijs and De Clercq 2002, 2004; Ferkovich and Shapiro 2004; Bonte and De Clercq 2008).

To evaluate the quality of insects, several biological parameters such as immature developmental time and survival, body weight, fecundity, and longevity are routinely used (Grenier and De Clercq 2003). In many synovigenic insects, determining lifetime fecundity is a tedious and time-consuming activity. Bonte and De Clercq (2008) proposed a method to assess fecundity of *O. laevigatus* based on oocyte counts in dissected female adults. In the latter study, oocyte counts at day eight were strongly correlated with lifetime oviposition of the predator reared on different diets. Similar methods have been developed for *Macrolophus* spp. by Callebaut et al. (2004) and Vandekerkhove et al. (2006).

In the present study, we hypothesized that both *O. thripoborus* and *O. naivashae* are amenable to mass production and may have potential as biological control agents in southern Africa. First, the effect of moisture source on the development of *O. thripoborus* and *O. naivashae* was determined. Second, the impact of several factitious foods and bee pollen on developmental and reproductive parameters of both *Orius* species was studied. Finally, the reliability of the dissection test designed by Bonte and De Clercq (2008) to predict the influence of diet on the reproductive potential of *O. laevigatus* was investigated for these two little-studied *Orius* species from southern Africa.

## Materials and Methods

### *Orius thripoborus* and *O. naivashae* collection and stock cultures

Nymphs and adults of *O. thripoborus* were collected in August and September 2008 in and around sugarcane (*Saccharum officinarum*) fields in the South African provinces Mpumalanga and KwaZulu-Natal. A colony of *O. naivashae* was initiated in 2009 with nymphs and adults collected from flowering weeds in the vicinity of sugarcane fields in KwaZulu-Natal, South Africa. Specimens were identified using the keys developed by Hernández and Stonedahl (1999), and their identities were confirmed by Dr. Berend Aukema.

Both stock colonies were cultured in Plexiglas containers (9 cm diameter, 4 cm high) as described by Van de Veire (1995). Each cage contained a sharp pepper plant (*Capsicum annuum*) as a moisture source and oviposition substrate. Adults were fed a mixture of *E. kuehniella* eggs and dry honey bee pollen (N.V. Weyns's Honingbedrijf, www.weyns-honing.com). To prevent cannibalism, a wrinkled piece of wax paper (5 × 10 cm) was placed in each container (Bonte and De Clercq 2010b). The rearing cages were maintained in growth chambers at 25 ± 1 ^°^C, 65 ± 5% RH and a photoperiod of 16:8 L:D. All experiments were conducted in an incubator under the same conditions but at a temperature of 23 ± 1 °C.

### Influence of water source on development

In a first experiment, the effect of two water sources on the developmental performance of *O. thripoborus* and *O. naivashae* was assessed. In the first treatment, water was provided in hemispherical domes (70 µL) made of Parafilm M using an encapsulation device (Analytical Research Systems, Inc., www.ars-fla.com). Stretching the Parafilm M before encapsulation facilitated stylet penetration by early instars of the insect. The domes were sealed using transparent Scotch 3M Packaging Super Tape (3M, www.3m.com). In the second treatment, a flat green bean pod (*Phaseolus vulgaris*) was used as a source of water. Green bean pods were thoroughly washed before being used in the experiment to avoid contamination with pesticide residues. The bean pod was cut between two seeds into 2–3 cm pieces to prevent nymphs from hiding inside the bean. As this plant substrate also provides nutrients besides being a source of moisture, ‘water source’ is an operational term and is not meant to be physiologically defining. Frozen eggs of *E. kuehniella* were supplied as food in both treatments. *Ephestia kuehniella* eggs and water sources were refreshed every other day.

For each treatment, 40 first instars (< 24 hours old) were caged in individual plastic containers (4.5 cm diameter, 3 cm high) sealed with a lid having a ventilation hole covered with a fine mesh gauze. Development and survival of nymphs were monitored daily, and newly emerged adults were sexed and weighed using a Sartorius Genius ME215P balance (Sartorius, www.sartorius.com).

A two-way ANOVA was conducted to evaluate whether water source had a different effect on developmental time and body weights of adult males and females of *O. thripoborus* and *O. naivashae.* As no interaction occurred between the main factors water source and species for all tested parameters, means within each factor were separated using the Tukey pairwise comparison procedure (Kutner et al. 2005). Survival rates were compared by means of a logistic regression. This regression is a generalized linear model using a probit (log odds) link and a binomial error function. Each test consists of a regression coefficient that is calculated and tested for being significantly different from zero, for which *p*-values are presented (McCullagh and Neider 1989). *p-*values smaller than or equal to 0.05 are considered significant. Sex ratios were evaluated versus an equal male: female distribution (1:1 ratio) by means of a nonparametric Chi-Square test (SPSS-Inc. 2006).

### Effect of diet on development and reproduction

**Diets.** In a second experiment, three foods were tested on both *Orius* species: two factitious prey types and one plant diet. The first factitious food consisted of frozen eggs of *E. kuehniella,* which were supplied by Koppert Biological Systems (www.koppert.com). A second factitious food consisted of hydrated decapsulated cysts of the brine shrimp *Artemia franciscana* Kellogg (Crustacea: Artemiidae), originating from Great Salt Lake, Utah, USA, and supplied by the *Artemia* Reference Center at Ghent University in Ghent, Belgium). The cysts were hydrated by placing them in tap water for two hours, after which excess water was removed. The plant diet was composed of moist frozen honey bee pollen, also supplied by Koppert Biological Systems. The pollen pellets were finely crushed before being offered to the predator.

In each treatment, a flat green bean pod was provided as a water source, substrate (hiding place), and extra nutrient source. All foods were supplied ad libitum and replenished every other day, except for *A. franciscana* cysts, which were refreshed on a daily basis.

**Nymphal development.** For each diet, 120 first instars (< 24 hours old) of both *Orius* species were individually caged in 5 cm diameter containers. Developmental performance of the predators was assessed as described above.

A two-way ANOVA was conducted to evaluate effects of diet on developmental time and body weight of adult males and females of both predators. Where no interaction was found, means were separated using a Tukey test. When interactions were significant, pairwise multiple comparison procedures were used (Kutner et al. 2005). When a Kolmogorov—Smirnov test indicated that these means were normally distributed, the parameter was analyzed using a one-way analysis of variance (ANOVA). When means were not normally distributed, a non-parametric Kruskal-Wallis H test was used. Survival rates were compared by means of a logistic regression (SPSS-Inc. 2006).

### Reproduction

For each diet, newly emerged adults (< 24 hours old) were paired and transferred into 5 cm diameter containers. The adults were offered the same diet as in their nymphal life. Half of the females were dissected, whereas the other half was held to determine lifetime oviposition. The latter group of females were offered a piece of green bean pod as an oviposition substrate. The bean pods were checked daily for eggs to determine the preoviposition period. When the first egg was laid, bean pods were replaced every other day until the female died. Lifetime oviposition and egg hatch were monitored. Eight days after adult emergence, the second half of the females was dissected to quantify oocyte development. For dissection, the females were pinned down on their dorsal side. The ovipositor together with the last two abdominal segments was carefully separated from the abdomen, exposing the ovaries. The number of oocytes (follicles) in the ovaries and oviducts was counted and scored according to the method described by Callebaut et al. (2004): late vitellogenic to mature oocyte, 1; early to mid vitellogenic oocyte, 0.5; previtellogenic oocyte, 0.25; no observable oocyte, 0; early previtellogenic oocytes that were not clearly discernible under the dissection microscope (magnification 25×) were not scored. During the eight-day period before dissection, oviposition of this cohort was monitored. As the presence of an oviposition substrate could affect oocyte counts for this cohort, the bean pod was replaced by a water dome to provide moisture. Whereas Shapiro and Ferkovich (2006) used water-filled Parafilm domes to collect *O. insidiosus* eggs, females of *O. laevigatus* (Bonte and De Clercq 2010a), *O. thripoborus,* and *O. naivashae* were rarely observed to deposit eggs into water domes.

To evaluate whether diet had a different effect on reproduction of *O. thripoborus* and *O. naivashae*, measures of reproduction were subjected to a two-way ANOVA. As no interaction between diet and species was found for the parameters preoviposition period, lifetime oviposition, weighted sum of oocytes, and longevity, means within each factor were separated using the Tukey pairwise comparison procedure (Kutner et al. 2005). Means for egg hatch were compared by way of a logistic regression. To evaluate the relationship between lifetime fecundity and oocyte counts, a Pearson's correlation test was performed (SPSS-Inc. 2006).

## Results

### Influence of water source on development

Nymphal survival was significantly affected by water source but not by species (Table 1). Survival rate of nymphs of both *O. thripoborus* and *O. naivashae* fed *E. kuehniella* eggs was about 30% higher when a bean pod was offered as a water source than when a water dome was offered (Table 2).

Both male and female developmental time was influenced by species. Regardless of water source, *O. thripoborus* developed faster than *O. naivashae* (Tables 1 and 2). Male developmental time was also influenced by water source. When a bean pod was offered, males of both *O. thripoborus* and *O. naivashae* developed faster than when a water dome was offered (Table 1).

Neither of the tested factors influenced adult weight of both males and females (Table 1).

Sex ratios of both species within all treatments did not deviate significantly from a 1:1 ratio (Table 2), although *O. naivashae* produced more females than males in both treatments.

### Effect of diet on development and reproduction

**Nymphal development.** Table 3 presents the results of a two-way ANOVA assessing the effect of diet and species on developmental parameters.

Regardless of the species, nymphal survival of predators fed *E. kuehniella* eggs was not significantly different from that of predators reared on *A. franciscana* cysts (logistic regression; *p* > 0.377). On the other hand, nymphal survival of the anthocorids fed either factitious food (*E. kuehniella* eggs or *A. franciscana* cysts) was significantly better than that of those fed pollen (*p* < 0.01 in both cases). For both anthocorids, nymphal survival ranged from 66.3 to 86.6% on the tested diets (Table 4).

For developmental time, interactions were found to be significant (two-way ANOVA); here, the treatment means were compared pairwise using multiple comparison procedures. As developmental times were not normally distributed they were analyzed using a non-parametric Kruskal-Wallis H test.

Development of males (χ^2^ = 144.843; df = 5; *p* < 0.01) and females (χ^2^ = 244.275; df = 5; *p* < 0.01) of either species was faster on the factitious foods than on pollen. Predators fed flour moth eggs had shorter developmental times than those fed brine shrimp cysts (Table 4). When pollen was offered as food, development took longer than when cysts were offered, except for *O. naivashae* males. In the latter case, no significant differences in developmental time were observed between pollen and *A. franciscana* cysts.

As there was no interaction between diet and species for weights of male adults (two-way ANOVA), means within each factor were separated using a Tukey test.

Both diet and species influenced male adult weight. In general, *O. naivashae* males were heavier than *O. thripoborus* males (Table 3). *Artemia* cysts and pollen yielded males with similar adult weights (*p* = 0.295) which were in turn lighter than those reared on *E. kuehniella* eggs (*both p* < 0.01).

For female adult weight, interactions were found to be significant (two-way ANOVA). Consequently, the treatment means were compared pairwise using multiple comparison procedures; here, body weights of female adults were normally distributed and therefore analyzed using a one-way analysis of variance (ANOVA). Their variances of means were heteroscedastic and hence separated using a Tamhane test (*p =* 0.05).

Female adult weights of *O. thripoborus* were similar to those of *O. naivashae* on cysts and pollen, but adults of the latter species produced on *E. kuehniella* eggs were heavier than those of the former (one-way ANOVA; *F =* 33.881; df = 5, 336; *p* < 0.01). Within *O. naivashae,* flour moth eggs yielded heavier females than brine shrimp cysts, which in turn yielded heavier females than did pollen (Table 4).

Sex ratios of *O. naivashae* were female biased on all diets (*p* < 0.01). For *O. thripoborus,* no significant deviations from a 1:1 sex ratio were observed (Table 4).

### Reproduction

Reproduction characteristics of both *Orius* species reared on different diets are given in Table 6. Females of both species were able to produce viable eggs on all diets.

The diet × species interaction was only significant for the parameter egg hatch (Table 5). Egg hatch exceeded 83% in all treatments. Hatching rate of *O. thripoborus* eggs was superior to that of *O. naivashae* eggs except on *A. franciscana* cysts. For *O. thripoborus, E. kuehniella* eggs resulted in the highest egg hatch, whereas *A. franciscana* cysts yielded the lowest hatching rate. In *O. naivashae,* egg hatch did not differ among diets (Table 6).

Preoviposition period was affected by species but not by diet. Females of *O. thripoborus* had shorter preoviposition periods than those of *O. naivashae* (Table 5).

Both diet and species influenced lifetime oviposition. Regardless of diet, *O. thripoborus* produced more eggs than *O. naivashae* (Tables 5 and 6). Variability of lifetime oviposition was high, with coefficients of variation ranging from 70.7 to 89.3%. Overall, females of both species fed pollen laid 26–51% of the number of eggs deposited by those fed *E. kuehniella* eggs or *A. franciscana* cysts (Table 6).

Oocyte counts were similar in all treatments (Table 5) and varied between 7.6 and 11.2 (Table 6).

A strong significant correlation was found between lifetime oviposition and the weighted sum of oocytes at dissection for *O. thripoborus* (r = 0.999; *p* < 0.05; n = 3), but for *O. naivashae* the correlation was not significant at the 0.05 level, despite a high magnitude of the correlation coefficient (r = 0.992; *p* > 0.05; n = 3).

Longevity of females varied between 50.5 and 61.1 days and did not differ among treatments (Tables 5 and 6). Male longevity depended on both diet and species. In general, *O. naivashae* males lived longer than those of *O. thripoborus* (Table 5). Regardless of the species, males lived longer on *E. kuehniella* eggs than *on A. franciscana* cysts (*p* < 0.05).

## Discussion

The only records for *O. thripoborus* in South Africa were made on citrus (*Citrus* sp.) (Hesse 1940) and avocado (*Persea americana*) (Steyn et al. 1993), both preying on thrips species damaging the fruits. Our survey yielded the first record of *O. naivashae* in South Africa. During our sampling in and around sugarcane fields, *O. naivashae* was only found in the more temperate southern part, preying on thrips in flowers of *Senecio madagascariensis, Ageratum conyzoides,* and *Bidens pilosa* (all Asteraceae). Only on a few occasions were *O. naivashae* found in the inflorescences of sugarcane. On the contrary, *O. thripoborus* was recovered in sugarcane flowers as well as in neighboring flowering weeds, and was recorded at more sampling sites than *O. naivashae.*

In a preliminary experiment, only a single *O. thripoborus* nymph (out of 20) reached the adult stage when *E. kuehniella* eggs were offered without a supplementary water source. Despite the ∼68 % water content of *E. kuehniella* eggs (De Clercq et al. 2005), a supplementary source of water was needed to sustain the development of the predator when presented with this food. In most rearing systems for heteropteran predators, water is supplied via plant materials. Besides being a source of moisture, plants also serve as an oviposition substrate (Castañé and Zalom 1994; Coll 1996; Richards and Schmidt 1996; Lundgren and Fergen 2006) and provide hiding places, thus reducing cannibalism (Van de Veire 1995; Cocuzza et al. 1997b). In addition, it has been shown that several heteropteran predators, including *Orius* spp., may also derive supplemental nutrients from plant materials (Lundgren 2009). *Orius insidiosus* gains water from the plant xylem, and may ingest small amounts of starches, sugars, and amino acids from the mesophyll (Armer et al. 1998). The influence of plant materials on the performance of predatory bugs varies greatly (Naranjo and Gibson 1996; Lundgren 2009). Overall, supplementing prey diet with plant material has been reported to accelerate nymphal development, increase nymphal survival and adult longevity, and enhance fecundity (Coll 1998). In our experiments, nymphal survival was higher and development of males faster when a piece of bean pod was added as water source to *E. kuehniella* eggs, for both *O. thripoborus* and *O. naivashae.* Richards and Schmidt (1996) stated that bean pods were an important source of moisture, greatly affecting the proportion of nymphs of *O. insidiosus* reaching the adult stage. Kiman and Yeargan (1985), on the other hand, observed no differences in nymphal survival or developmental time of this species when a bean pod was supplemented to *Heliothis virescens* eggs. Bush et al. (1993) noted a faster development and better fecundity but similar survival when *O. insidiosus* were offered a bean pod in addition to *H. virescens* eggs. In contrast, nymphs of *O. laevigatus* developed slower when a bean pod was added to a diet of *E. kuehniella* eggs as compared with free water as a moisture source (Bonte and De Clercq 2010b).

Our findings suggest that the presence of the bean pod had a positive influence on some of the developmental parameters of the tested *Orius* species. Bonte and De Clercq (2010a) pointed out that the use of plant materials to provide moisture in rearing systems for predatory heteropterans has several drawbacks. Artificial sources of water, like the Parafilm domes used in our study, may contribute to rationalizing the rearing process. However, for some species it may be advisable to compensate for the extra nutrients, which are normally gained from plant materials, when only free water is provided.

Several studies have shown that eggs of the Mediterranean flour moth *E. kuehniella* constitute a nutritionally superior food for *Orius* bugs (e.g., Cocuzza et al. 1997a; Ferkovich and Shapiro 2004; Bonte and De Clercq 2008). Arijs and De Clercq (2001) and Bonte and De Clercq (2008) demonstrated that hydrated decapsulated cysts of *A. franciscana* also sustained development and reproduction of *O. laevigatus,* with similar or slightly inferior results as compared with *E. kuehniella* eggs. The current study indicates that *Artemia* cysts were also an acceptable food for *O. thripoborus* and *O. naivashae* and may thus have value for use in the mass production of these species as well. However, *Artemia* cysts may not be a suitable food to solely support long term cultures of *Orius* spp. (De Clercq et al. 2005) and may thus have more potential to replace the more expensive *E. kuehniella* eggs in part of the rearing process.

*Orius thripoborus* and *O. naivashae* were able to complete their development on moist honey bee pollen. With nymphal survival percentages of 66% for *O. thripoborus* and 78% for *O. naivashae,* mortality on honey bee pollen was higher than on the tested factitious foods. However, these survival rates are similar or even better than those reported for other *Orius* spp. reared on pollen from various sources in combination with plant tissue (Kiman and Yeargan 1985; Richards and Schmidt 1996; Vacante et al. 1997; Venkatesan et al. 2008; Lundgren 2009; Bonte and De Clercq 2010b).

Overall, developmental fitness of both tested *Orius* species on factitious foods was better than on pollen. There is considerable variation in the performance of *Orius* nymphs on pollen among studies. Reported developmental times of different *Orius* spp. are generally longer on vegetal diets (e.g., pollen) than on insect prey (e.g., Kiman and Yeargan 1985; Funao and Yoshiyasu 1995; Richards and Schmidt 1996). Lundgren (2009) showed that pollen from certain hybrids of *Zea mays* did not support development in *O. insidiosus* at all, whereas that from others only allowed a small number of individuals to complete development. In contrast, Duan et al. (2007) reported good survival and rapid development of *O. insidiosus* on a bee pollen diet consisting of 40% water and 60% pollen. They suggested the use of this pollen diet in Tier-I toxicity assays to evaluate potential adverse effects of transgenic plants on non-target heteropteran predators. Females of *O. thripoborus* and *O. naivashae* showed a reduction in fecundity of 54 and 74%, respectively, when fed on pollen, as compared to when fed on *E. kuehniella* eggs. Other studies reported a strong reduction in fecundity for *Orius* spp. fed pollen instead of prey (Fauvel 1974; Salas-Aguilar and Ehler 1977; Kiman and Yeargan 1985; Cocuzza et al. 1997b). Females of *O. tantillus* failed to lay any eggs when reared on maize pollen (Venkatesan et al. 2008). Differences in developmental and reproductive performance of *Orius* spp. feeding on pollen could be due to differences in nutritional quality, particularly pertaining amino acid and lipid content, and defensive properties of the pollen (Stanley and Liskens 1974; Richards and Schmidt 1996). Besides defensive structural traits of pollen grains, their antinutritive or even toxic qualities may have a negative impact on the biological performance of pollinivores (Lundgren 2009).

Despite that animal prey is required for optimal development and reproduction, numerous workers have observed *Orius* spp. feeding on pollen in the field (e.g., Salas-Aguilar and Ehler 1977; Cocuzza et al. 1997b; Corey et al. 1998). Pollinivory is considered to be an adaptive strategy to sustain populations of these predators when prey numbers are low, which may eventually lead to a more effective pest control. Feeding on pollen may also play an important role in a preventive release strategy (Cocuzza et al. 1997b). Populations of *Orius* spp. may be supported by pollen-producing wild or cultivated plants in the vicinity of the crop. Alternatively, the pollen itself can be applied to the crop (e.g., van Rijn et al. 2002). However, the outcome of conservation measures based on increasing pollen input may not be unequivocal. Skirvin et al. (2007) found that the presence of pollen reduced predation by *O. laevigatus* of thrips by 40%, leading to higher pest populations. More on the negative side, the plant feeding habit may expose *Orius* bugs to systemic insecticides (Cocuzza et al. 1997b).

Like in *O. laevigatus* (Bonte and De Clercq 2008), lifetime oviposition data and oocyte counts were strongly correlated in *O. thripoborus* and *O. naivashae* females reared on different diets. Some caution is warranted in the case of *O. naivashae,* for which the correlation was only marginally significant, due to the high variability of the oviposition data. This linear relationship may thus be used to more cost-effectively predict the reproductive capacity of these predators as a function of their diet (Bonte and De Clercq 2008).

Whereas reported sex ratios in other *Orius* spp. are essentially 1:1, sex ratios of *O. naivashae* were female biased, particularly in the second experiment where 2 to 4.5 times more females emerged than males. A similar trend was observed in the stock culture of *O. naivashae.* Sex-determining mechanisms in invertebrates are of genetic and/or environmental origin, but also cytoplasmic factors (like the endosymbionts *Wolbachia* and *Spiroplasma*) might be involved (Stouthamer et al. 1999; Cook 2002). As nymphal survival in our experiments was very high, skewed sex ratios are unlikely the result of differential survival of males and females. The only other report of a skewed sex ratio in an *Orius* sp. is that by Shapiro et al. (2009), where a skewed field sex ratio was found for *O. insidiosus* in the favor of males, which was most likely the result of sampling error or differential hatch rate or survival of one sex.

Our findings indicate that both *O. thripoborus* and *O. naivashae* are easily produced in the laboratory using factitious foods. Developmental and reproductive performance of *O. thripoborus* was superior to that of *O. naivashae,* with a faster nymphal development, shorter preoviposition period, and overall better fecundity. *Orius thripoborus* also performed better on pollen than *O. naivashae.* Body weights of both species in our study were generally similar, except when the predators were offered *E. kuehniella* eggs and green beans, resulting in heavier body weights for *O. naivashae.* In field collections, adults of *O. naivashae* are mostly larger than those of *O. thripoborus* (Hernández and Stonedahl 1999). However, it is not known if the larger size of *O. naivashae* is beneficial in terms of predation capacity as compared with *O. thripoborus.* Our first findings may lead to the conclusion that *O. thripoborus* has greater potential than *O. naivashae* for use in biological control programs. However, it deserves emphasis that in the current study the predators were reared individually and only so for one generation under (optimal) laboratory conditions. Other factors like diapause (Chambers et al. 1993; van den Meiracker 1994), temperature preferences, searching behavior, predation capacity (Coll and Ridgway 1995), and habitat and prey preference (Honda et al. 1998) may determine the effectiveness of these predators in the field. Therefore, further study is warranted to elucidate the ecology and biocontrol potential of these and other little-studied African species of the genus *Orius.*

**Table 1.**
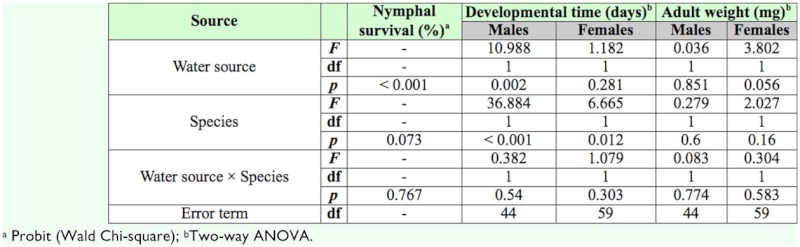
Results of a logistic regression and a two-way ANOVA indicating the effect of water source (water dome or bean pod) and species (*Orius thripoborus* and *Orius naivashae*) on developmental parameters.

**Table 2.**
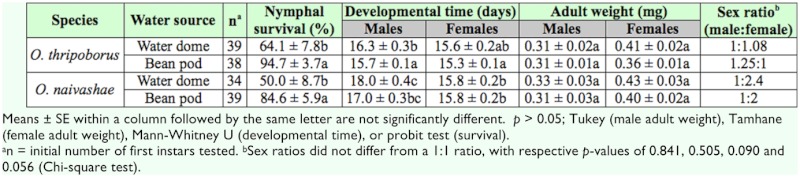
Developmental parameters of *Orius thripoborus* and *Orius naivashae* on two water sources.

**Table 3.**
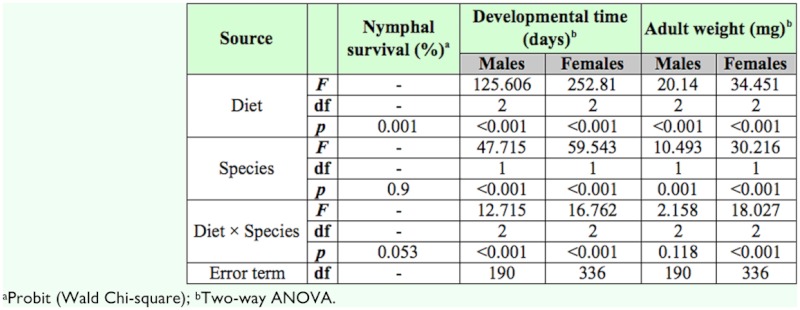
Results of a logistic regression and a two-way ANOVA indicating the effect of diet (*Ephestia kuehniella* eggs, *Artemia franciscana* cysts or bee pollen) and species (*Orius thripoborus* and *Orius naivashae)* on developmental parameters

**Table 4.**
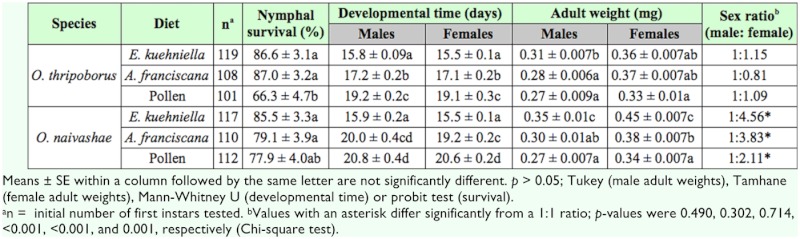
Developmental parameters of *Orius thripoborus* and *Orius naivashae* on three diets.

**Table 5.**
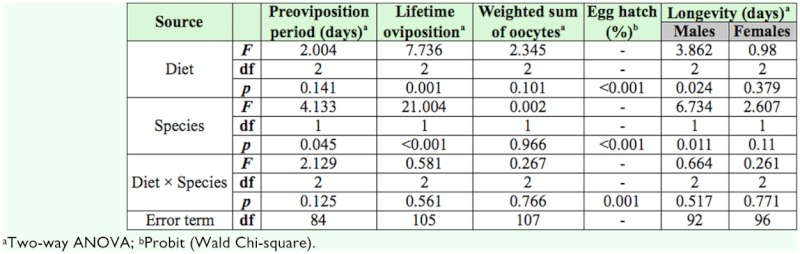
Results of a logistic regression and a two-way ANOVA indicating the effect of diet and species (*Orius thripoborus* and *Orius naivashae*) on reproductive parameters.

**Table 6.**
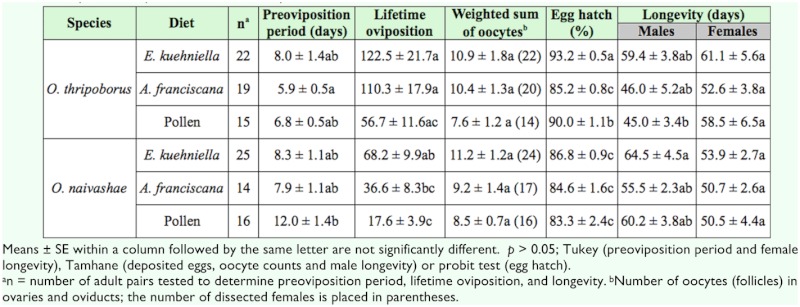
Reproductive parameters of *Orius thripoborus* and *Orius naivashaeon* three diets.
